# Association of X-linked TLR-7 gene polymorphism with the risk of knee osteoarthritis: a case–control study

**DOI:** 10.1038/s41598-022-11296-4

**Published:** 2022-05-04

**Authors:** Xutao Xi, Arshad Mehmood, Pengyan Niu, Jinjie Yang, Yintian Wang, Heyu Zhou, Xiaohui Han, Lifen Ma, Shiying Jin, Yinxia Wu

**Affiliations:** 1 Department of Orthopedics, Handan First Hospital, Handan, 056000 Hebei China; 2 Department of Neurology, Handan First Hospital, Handan, China; 3 Department of Neurology, The Second Hospital of Hebei Medical University, Shijiazhuang, Hebei China

**Keywords:** Genetics, Molecular biology

## Abstract

Knee osteoarthritis (OA) is the most prevalent type of OA, and Toll-like receptor 7 (TLR7) may lead to the pathogenesis of OA. Recently, X-linked TLR7 polymorphism has been confirmed to be associated with arthritis. However, there is a lack of studies on TLR7 gene polymorphism associated with knee OA susceptibility. The current study aimed to determine whether TLR7 gene polymorphism is associated with the risk of knee OA. Genotyping of two polymorphic sites (rs3853839 and rs179010) in the TLR7 gene was performed in 252 OA patients, and 265 healthy controls using the SNaPshot sequencing technique. Data were analyzed statistically by Chi-square tests and logistic regression. Rs3853839-C allele showed frequencies of 28% and 27% in the healthy control and female knee OA groups, respectively. The differences were not statistically significant (*P* > 0.05). The rs3853839-CG genotype frequency was significantly lower in the female knee OA group as compared to the healthy control group (OR 0.60; 95%CI 0.36–0.99; *P* = 0.044). In the male hemizygote population, the rs3853839-CC showed significantly lower frequencies in the male knee OA group as compared to the healthy control group (OR 0.35; 95%CI 0.17–0.71; *P* = 0.0025). Regarding rs179010, there were no differences in the genotype distribution and allele frequencies between OA patients and healthy subjects under any models (*P* > 0.05). Stratified analysis showed that the frequency of the rs3853839-CG genotypes was lower in high Kellgren-Lawrence grades (KLG) (OR 0.48; 95%CI 0.21–1.08; *P* = 0.066), and significantly lower in OA patients with effusion synovitis (OR 0.38; 95%CI 0.17–0.88; *P* = 0.013). TLR7 rs3853839 polymorphism may play a role in the susceptibility of knee OA in the Chinese Han Population and may be associated with OA severity and the risk of effusion synovitis in Knee OA.

## Introduction

Osteoarthritis (OA) is a degenerative joint disease that is the most common cause of disabilities among the aging population all over the world^1^. The incidence of OA increases by age and further increases with a longer lifetime. In the old population, symptomatic knee OA occurred more frequently in females than males^2^. Previous studies have shown that for elderly Chinese women, the prevalence of knee OA was higher than in Caucasian women^3^. The phenomenon may be related to ethnics and genetic factors.

However, the OA pathogenesis has not been fully understood. Arthrodial cartilage degradation and subchondral bone remodeling represent the most predominant pathological changes of OA^4^. The activation of innate immunity plays a critical role in the development and progression of OA. In the last few decades, TLR-mediated pro-inflammatory signaling pathway and their downstream nuclear factor-κB (NF-κB) signaling pathway have been extensively studied in OA^5–9^. Among TLR family members, TLR7 is the master regulator of RNA-driven TLR-dependent systemic autoimmune manifestations^10^. Previous studies revealed that TLR7 expression was elevated and may be identified for OA diagnostic^11^. Moreover, the TLR7 signaling pathway may play a vital role in OA, and the TLR7 antagonist also exerts a long-lasting analgesic effect on knee OA pain^12^.

TLR7 gene is located on the X-chromosome^13^, which carries multiple polymorphisms potentially associated with human disease, including susceptibility and progression of HIV-1 infection, asthma, autoimmune thyroiditis, systemic lupus erythematosus (SLE)^14–17^. The rs3853839 C/G single-nucleotide polymorphism (SNP), which is located in the 3′ untranslated region of the TLR7, has been associated with an increase in TLR7 mRNA and protein expression, as well as upregulation of idiopathic subglottic stenosis (iSGS)^18–20^. In addition, rs179010 as an intronic polymorphism has already been reported to be associated with hand, foot, and mouth disease caused by enterovirus 71 in male children and SLE^21,22^. Polymorphisms in TLR genes have been associated with OA^23–25^. However, no relevant pieces of literature report the relationship between X-linked TLR7 gene polymorphism and the risk of knee OA.

In this case–control study, we identified X-linked TLR7 3′ UTR gene polymorphism and intronic polymorphism with the risk of knee OA.

## Materials and methods

### Subjects

OA patients were recruited at the Department of Orthopedics, Handan First Hospital, between May 2018 and May 2020, while healthy controls were enrolled from the physical examination center. All recruited subjects were from the northern Chinese population in the Handan city of Hebei province. Patients were recruited if they were diagnosed with knee OA, and fulfilled the American College of Rheumatology criteria^26^. As a result, patients who met the following criteria were excluded: (a) diagnosed with hypertension, diabetes, cerebrovascular disease, tumors, and other chronic diseases; and (b) complicated with other autoimmune diseases, such as rheumatoid arthritis and SLE, among others autoimmune diseases. All patients agreed to participate in the study and signed a written informed consent before enrolment. Patient data, including age, gender, clinical manifestations, smoking, drinking, body mass index (BMI), MRI of the knee, and K-L grading, were collected and entered into a standard case report form. The study was approved by the Ethics Committee of Handan First Hospital. All methods were performed following the Declaration of Helsinki guidelines and regulations.

### DNA isolation and genotyping

DNA was isolated from each patient and subjected to SNaPshot genotyping. Briefly, The peripheral venous blood (ca. 5 mL) was collected into tubes containing EDTA as an anticoagulant. Genomic DNA was extracted from the samples using the Blood Genomic DNA Extraction Kit (JieRui, Shanghai, China). The DNA was subjected to PCR using PCR MIX (Yisheng Biotech Co., Shanghai, China), targeting the SNPs. The primer's information were shown in Table 1.

**Table 1:** Primer information and primer sequences of TLR7.

Genes	Upstream primer	Downstream primer	Extension primer
rs3853839	CCATTCTGTGC	CTTTCTTTCTTACT	CTGACTGGGAACCC
	AGATTGAG	GTTTCCCTA	AGAAGCAGGCCCAAG
rs179010	AGTTATACGGTT	CCTACACATTGGG	TCCTCTGATTATGTA
	CCTCTGATTA	TTTACATT	TCACC

### Statistical analysis

The online software SNPStats (https://www.snpstats.net/start.htm) and the SHEsis (http://analysis.bio-x.cn) were used to construct linkage disequilibrium (LD), Hardy–Weinberg balance test haplotypes, and analyzed the interactions with related factors, while the other statistical analyses were performed using SPSS version 21.0 (IBM Corp., Armonk, NY). Results were presented as frequencies (percentages) for qualitative variables and mean ± standard deviations (SD) for quantitative variables. Comparisons between two groups were performed using unpaired student's t-test, whereas Chi-squared tests were used for categorical variables. The association between SNPs with the risk factors was analyzed using logistic regression analysis data followed by (*P* < 0.05) were considered statistically significant. The computed statistical power for this study was 0.99 (n = 517, P0 = 0.2, α = 0.01, OR 2) using PASS 15.0.

## Results

### Analysis of clinical data

A total of 252 OA patients (146 females and 106 males, respectively) and 265 healthy controls (158 females and 108 males, respectively) were enrolled. A summary of patient characteristics is presented in Table 2. There were no statistically significant differences between the case and control groups concerning age, smoking, and drinking distribution (*P* > 0.05). OA patients showed a slightly higher, but insignificant, mean BMI (24.3 ± 2.21) as compared to controls (24.0 ± 1.92) (*P* = 0.088). The KLG 2, 3, and 4 were identified in 63, 97, and 92 OA cases, respectively. Moreover, Knee magnetic resonance imaging (MRI) assessed effusion synovitis damage using semiquantitative whole-organ MRI scoring (WORMS). There were 32, 23, 45, and 25 cases for WORMS (0–3), respectively. The results are shown in Table 2.

**Table 2:** Demographics and clinical characteristics of participants for TLR7 SNP.

Clinical characteristics	Knee OA	HC	*P* values
	n = 252	n = 266	
Sex			0.4916
Females	146	158	
Males	106	108	
Age, y (mean ± SD)	56 ± 15	55 ± 11	0.372
Smoking			0.075
Yes	71	57	
No	118	209	
Drinking			0.392
Yes	57	52	
No	195	214	
Body mass index (kg/m^2^)	24.3 ± 2.21	24.0 ± 1.92	0.088
Kellgren–Lawrence grade			
2	63	NA	NA
3	97	NA	NA
4	92	NA	NA
MRI of knee WORMS score			
0	32	NA	NA
1	23	NA	NA
2	45	NA	NA
3	25	NA	NA

*Knee OA* knee osteoarthritis patients group, *HC* healthy control group, *SD* standard deviation, *NA* not assessable, *WORMS* Semiquantitative whole-organ MRI scoring.

### SNP selection and genotyping

Two loci were detected on the TLR7 gene (Table 3). Rs3853839 (C > G) located on chromosome X: 12889539 (GRCh38) and rs179010 (T > C) located on X: 12884766 (GRCh38) represented respectively 3′ UTR region mutations and intron region of TLR7. Genotypic distributions of rs3853839 and rs179010 were consistent with Hardy–Weinberg equilibrium in healthy controls (*P* > 0.05), suggesting that the selected sample population was representative. Because the TLR7 gene is located on the X chromosome, only female subjects were used to perform the Hardy–Weinberg equilibrium test. The results are shown in Table 3.

**Table 3:** Selected SNPs of TLR7 and PHWE in this study.

SNPs	Location	Group	Genotype (n)			PHWE	1000genome CHBs	Nucleotide	Functional region
rs3853839	X:12889539		CC	CG	GG		G = 0.40		
	(GRCh38)	Knee OA (n = 146)	87	40	19	*P* = 0.0005		C > G	3_prime_UTR
		HC (n = 158)	82	60	16	*P* = 0.34			
rs179010	X:12884766		CC	CT	TT		T = 0.29		
	(GRCh38)	Knee OA (n = 146)	78	50	18	0.045		T > C	Intron
		HC (n = 158)	78	59	21	0.098			

*Knee OA* osteoarthritis patients group, *HC* healthy control group, *PHWE P* value of Hardy–Weinberg equilibrium.

### Association between two sites and OA risk under different models

Since the TLR7 gene is located on the X chromosome, the gender distribution influences the allele frequency calculations. Gene frequencies were calculated for men and women separately. The genotype distributions and allele frequencies of the two polymorphisms are shown in Tables 4 and 5.

**Table 4:** Association analysis between SNPs and the female Knee OA risk under different models.

SNPs	Model	Genotype	HC (n = 158)	Knee OA (n = 146)	OR (95% CI)	*P*-value
rs3853839	Allele	G	224 (72%)	214 (73%)	1	0.50
		C	92 (28%)	78 (27%)	0.88 (0.62–1.26)	
	Codominant	G/G	82 (51.9%)	87 (59.6%)	1	0.13
		C/G	60 (38%)	40 (27.4%)	0.59 (0.35–0.99)	
		C/C	16 (10.1%)	19 (13%)	0.90 (0.39–2.07)	
	Dominant	G/G	82 (51.9%)	87 (59.6%)	1	0.072
		C/G-C/C	76 (48.1%)	59 (40.4%)	0.65 (0.40–1.04)	
	Recessive	G/G-C/G	142 (89.9%)	127 (87%)	1	0.84
		C/C	16 (10.1%)	19 (13%)	1.09 (0.48–2.45)	
	Overdominant	G/G-C/C	98 (62%)	106 (72.6%)	1	0.044
		C/G	60 (38%)	40 (27.4%)	0.60 (0.36–0.99)	
rs179010	Allele	C	215(0.68)	206(0.71)	1	0.50
		T	101(0.32)	86(0.29)	1.12(0.79–1.58)	
	Codominant	C/C	78 (49.4%)	78 (53.4%)	1	0.93
		C/T	59 (37.3%)	50 (34.2%)	0.97 (0.57–1.63)	
		T/T	21 (13.3%)	18 (12.3%)	0.86 (0.42–1.80)	
	Dominant	C/C	78 (49.4%)	78 (53.4%)	1	0.79
		C/T-T/T	80 (50.6%)	68 (46.6%)	0.94 (0.58–1.52)	
	Recessive	C/C–C/T	137 (86.7%)	128 (87.7%)	1	0.71
		T/T	21 (13.3%)	18 (12.3%)	0.88 (0.44–1.76)	
	Overdominant	C/C-T/T	99 (62.7%)	96 (65.8%)	1	0.99
		C/T	59 (37.3%)	50 (34.2%)	1.00 (0.60–1.64)	

*Knee OA* osteoarthritis patients group, *HC* healthy control group, *OR* odds ratios, *CI* confidence intervals. The data adjusted by smoke + drinking + age + BMI.

**Table 5:** Association analysis between SNPs and the male Knee OA risk.

SNPs	Genotype	HC (n = 108)	Knee OA (n = 106)	OR (95% CI)	*P*-value
rs3853839	G/G	75 (69.4%)	84 (79.2%)	1	0.0029
	C/C	33 (30.6%)	22 (20.8%)	0.35 (0.17–0.71)	
rs179010	C/C	96 (88.9%)	95 (89.6%)	1	0.7
	T/T	12 (11.1%)	11 (10.4%)	1.21 (0.47–3.11)	

*Knee OA* osteoarthritis patients group, *HC* healthy control group, *OR* odds ratios, *CI* confidence intervals; The data adjusted by smoke + drinking + age + BMI.

For rs3853839, C was the minor allele and showed frequencies of 28% and 27% in the control and female knee OA groups, respectively. The differences are not statistically significant. However, in the overdominant model, the rs3853839-CG genotype frequency was significantly lower in the female knee OA group as compared to the healthy control group (OR 0.60; 95%CI 0.36–0.99; *P* = 0.044). In the male hemizygote population, CC showed frequencies of 30.6% and 20.8% in the control and male knee OA groups, respectively. The differences are statistically significant (OR 0.35; 95%CI 0.17–0.71; *P* = 0.0025). For rs179010, there were no differences in the genotype distribution and allele frequencies between OA patients and healthy subjects under any models (*P* > 0.05). These findings indicated that TLR7 rs3853839 polymorphism may play a role in the susceptibility of knee OA in the Chinese Han Population.

### Linkage disequilibrium and haplotype analysis

**Figure 1:**
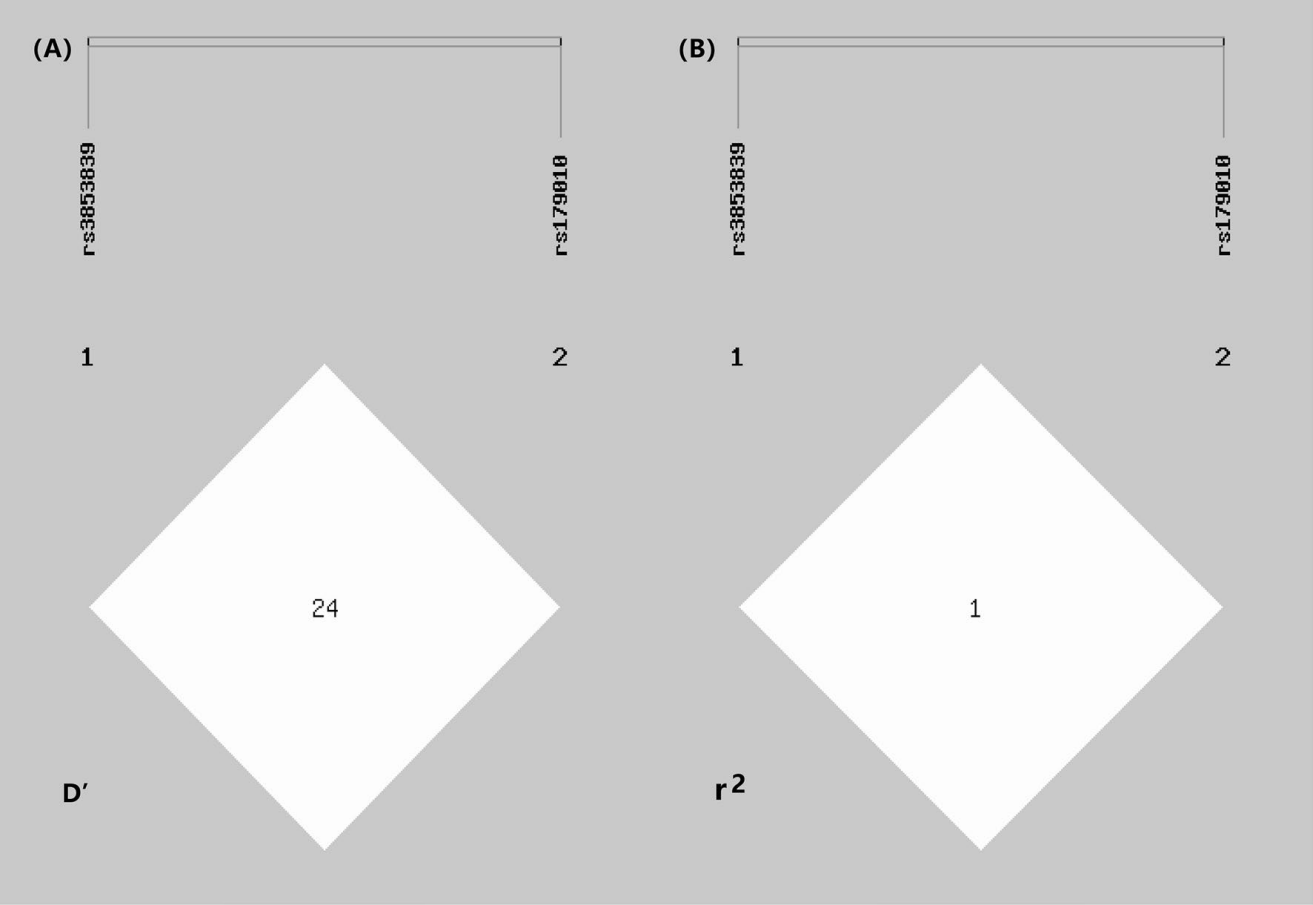
Linkage disequilibrium (LD) patterns in TLR7. D' (**A**) and r^2^ (**B**) mean LD coefficients of the two SNPs. Each block represents the LD relationship between two SNPs. Rs3853839 indicated no LD with rs179010.

LD was calculated from the female study sample. The haplotype analysis showed that rs3853839 and rs179010 were in low LD (D' = 0.2462). Thus, the haplotype analysis was not tested. The result was shown in Fig. 1.

### Stratification analysis based on clinical features of OA patients

No statistically significant difference was observed in genotype and allele frequencies after stratification by age, smoking status, and BMI. The association between TLR7 polymorphisms with OA severity was also calculated. Results showed the distribution of rs3853839-CG genotypes was lower in high KLG. The weight of evidence, while not achieving statistical significance, was towards a negative association (OR 0.48; 95%CI 0.21–1.08; *P* = 0.066), indicating that rs3853839 may be associated with OA severity (Table6).

**Table 6:** Stratified analyses based on clinical features for the association between TLR7 polymorphism and the risk of Knee OA.

SNPs	Genotype	Clinical features items		OR (95% CI)	*P*-value
		**BMI < 24**	**BMI ≥ 24**		
rs3853839	G/G-C/C	44 (77.2%)	62 (69.7%)	1	0.99
	C/G	13 (22.8%)	27 (30.3%)	0.99 (0.34–2.89)	
rs179010	C/C-T/T	39 (68.4%)	57 (64%)	1	0.2
	C/T	18 (31.6%)	32 (36%)	0.51 (0.18–1.44)	
		**Age > 55**	**Age ≤ 55**		
rs3853839	G/G-C/C	47 (69.1%)	59 (75.6%)	1	0.38
	C/G	21 (30.9%)	19 (24.4%)	0.72 (0.34–1.51)	
rs179010	G/G-C/C	47 (69.1%)	59 (75.6%)	1	0.38
	C/G	21 (30.9%)	19 (24.4%)	0.72 (0.34–1.51)	
		**KLG ≤ 3**	**KLG = 4**		
rs3853839	G/G-C/C	130 (81.2%)	82 (89.1%)	1	0.066
	C/G	30 (18.8%)	10 (10.9%)	0.48 (0.21–1.08)	
rs179010	C/C-T/T	125 (78.1%)	77 (83.7%)	1	0.2
	C/T	35 (21.9%)	15 (16.3%)	0.63 (0.31–1.29)	
		**Smoke = No**	**Smoke = Yes**		
rs3853839	G/G-C/C	135 (79.4%)	77 (93.9%)	1	0.53
	C/G	35 (20.6%)	5 (6.1%)	0.71 (0.24–2.08)	
rs179010	C/C-T/T	128 (75.3%)	74 (90.2%)	1	0.91
	C/T	42 (24.7%)	8 (9.8%)	0.95 (0.37–2.40)	

*Knee OA* osteoarthritis patients group, *HC* healthy control group, *OR* Odds ratios, *CI* confidence intervals, *KLG* Kellgren-Lawrence grade, *BMI* Body Mass Index. The data adjusted by moke + drinking + age + BMI.

We additionally dichotomized MRI-identified effusion-synovitis as none (WORSM 0) vs. any (WORSM 1–3) and investigated the relationship between the two SNPs on TLR7 and MRI-defined effusion-synovitis. For rs179010, no differences were observed in the genotype distribution frequencies between OA patients with effusion synovitis and healthy subjects (*P* > 0.05) under any models. However, the rs3853839 C/G + C/C genotype frequency in the effusion synovitis patient group under the dominant model was lower than that in the control group, and this difference was statistically significant (OR 0.56, 95%CI 0.33–0.95, *P* = 0.028). As well as, the C/G genotype frequency in the effusion synovitis patient group under the overdominant model was lower than that in the control group, and this difference was statistically significant (OR 0.48, 95%CI 0.24–0.97, *P* = 0.03). These results indicated that rs3853839 of TLR7 may be associated with the risk of effusion synovitis in Knee OA patients. (Table 7).

**Table 7:** Association between TLR7 polymorphism and the risk of effusion with synovitis in Knee OA.

SNPs	Model	Genotype	HC	Effusion synovitis	OR (95% CI)	*P*-value
rs3853839	Codominant	G/G	157 (59%)	64 (71.9%)	1	0.054
		C/G	60 (22.6%)	11 (12.4%)	0.45 (0.22–0.91)	
		C/C	49 (18.4%)	14 (15.7%)	0.70 (0.36–1.36)	
	Dominant	G/G	157 (59%)	64 (71.9%)	1	0.028
		C/G-C/C	109 (41%)	25 (28.1%)	0.56 (0.33–0.95)	
	Recessive	G/G-C/G	217 (81.6%)	75 (84.3%)	1	0.56
		C/C	49 (18.4%)	14 (15.7%)	0.83 (0.43–1.58)	
	Overdominant	G/G-C/C	206 (77.4%)	78 (87.6%)	1	0.03
		C/G	60 (22.6%)	11 (12.4%)	0.48 (0.24–0.97)	
rs179010	Codominant	C/C	174 (65.4%)	63 (70.8%)	1	0.57
		C/T	59 (22.2%)	18 (20.2%)	0.84 (0.46–1.54)	
		T/T	33 (12.4%)	8 (9%)	0.67 (0.29–1.53)	
	Dominant	C/C	174 (65.4%)	63 (70.8%)	1	0.35
		C/T-T/T	92 (34.6%)	26 (29.2%)	0.78 (0.46–1.32)	
	Recessive	C/C–C/T	233 (87.6%)	81 (91%)	1	0.37
		T/T	33 (12.4%)	8 (9%)	0.70 (0.31–1.57)	
	Overdominant	C/C-T/T	207 (77.8%)	71 (79.8%)	1	0.7
		C/T	59 (22.2%)	18 (20.2%)	0.89 (0.49–1.61)	

*Knee OA* osteoarthritis patients group, *HC* healthy control group, *OR* Odds ratios, *CI* confidence intervals; The data adjusted by smoke + drinking + age + BMI.

## Discussion

Elevated levels of TLR7 signaling molecules and their positive correlation with pain in OA patients suggest the involvement of the TLR7 signaling pathway in the pathogenesis of this disease^23–25^. In this regard, TLR gene polymorphisms have been reported to be associated with OA. However, the relationship between TLR7 gene polymorphism and the risk of knee OA has not been reported. This study aimed to explore the relationship between polymorphisms of the TLR7 gene with knee OA risk. The results showed that TLR7 rs3853839-CG frequencies of female OA subjects were significantly lower than that of healthy controls in the Chinese Han population. Furthermore, rs3853839-CC frequencies of male knee OA subjects were significantly lower than that of healthy controls. Moreover, rs3853839-CG genotypes were lower in high KLG, indicating that rs3853839 was associated with OA severity. Finally, the rs3853839 C/G + C/C genotype frequency in the effusion synovitis patient group was significantly lower than that in the control group. These results indicated that TLR7 rs3853839 polymorphism may play a role in the susceptibility of knee OA and may be associated with the risk of effusion synovitis in Knee OA patients.

Several studies identified that rs3853839 (G/C) as a 3′ UTR SNP in TLR7 is correlated with human disease^27^. Ming Yue et al. reported that TLR7 rs3853839-C allele with protection against HCV persistence in Chinese females^20^. The results of this study are consistent with the current study. In another study, Nan Shen et al. identified an association of rs3853839 (G/C) with SLE in 9,274 eastern Asians with a stronger effect in male than female subjects^19^. Moreover, one study reported in male participants that the reduced susceptibility to periodontitis was observed in carriers of TLR7-rs3853839-CC^28^. The results of this study supported our results. TLR7 rs3853839 polymorphism may play a role in the susceptibility of knee OA. The other SNP rs179010 was also reported to be related to human disease risk. In this respect, a study from Aya Kawasaki et al. reported that intronic SNPs rs179010 are associated with SLE independently of the 3′ UTR SNP rs3853839 in Japanese women^29^. Their study results showed rs3853839 and rs179010 were in moderate LD with each other^29^. Yaping Li et al. showed rs3853839 and rs179010 are correlated to the susceptibility and severity of hand, foot, and mouth disease caused by enterovirus 71 in male children^21^. In the current study, it was found that rs179010 gene polymorphism was not associated with knee OA risk. However, weak LD was also found between these two sites. The different results may be related to the patient selection biases.

Rs3853839, located in the 3′ UTR of the TLR7, has been confirmed to be functional and be associated with an increase in TLR7 mRNA and TLR7 protein expression^18–20^; thus, this was not confirmed by the present study. Moreover, results showed that rs3853839-CG genotypes carriers were found with mild-to-moderate KLG scores (1–3). This study also found the rs3853839 CG genotype frequency in the effusion synovitis patient was lower than that in the control group. It can be speculated that these sites might be functional SNP in the pathogenesis of knee OA. The 3′ UTR is an essential regulatory region for the expression of many genes that regulate the translation, degradation, and subcellular localization of the mRNAs by influencing with RNA-binding proteins or non-coding RNAs^30^. The 3′UTR polymorphisms might significantly impact gene expression by abolishing, weakening, or creating miRNA binding sites^31^. It has been shown that the non-risk C allele of rs3853839 matches a predicted binding site of microRNA-3148 (miR-3148), compared with the G-allele construct, the C-allele construct showed greater than two-fold reduction of luciferase activity in the presence of miR-3148^18^. However, more functional experiments should be needed for further verification.

Besides the TLR7 gene and other TLR genes polymorphisms have been associated with OA^23–25^. TLR7 gene expression and TLR7 signaling pathway may play a vital role in OA^11,13^. Together with the current study results, these clues have formulated a solid framework for further investigation of the mechanism of action of knee OA.

### Limitations

This study has several potential limitations which should be acknowledged. Despite expanding our knowledge of TLR7 regarding phenotype and genotype, there is no relevant research to confirm that these SNPs have functional effects. The role of TLR7 in OA has remained elusive. Furthermore, the sample size of the current study was small and obtained from a single center. Since only a few studies have reported the association between TLR7 rs3853839 and rs179010 polymorphism and knee OA risk, further investigations are required to verify the results.

## Conclusion

TLR7 rs3853839 polymorphism may play a role in the susceptibility of knee OA in the Chinese Han Population and may be associated with OA severity and the risk of effusion synovitis in Knee OA. This study provides a crucial basis for future research that explores the molecular mechanisms of the pathogenesis of knee OA.

## Data Availability

The data have been submitted to the European Variation Archive (EVA, https://www.ebi.ac.uk/eva/). The accessions associated with the data are Project: PRJEB51003, Analyses: ERZ5253848.
